# 基于亲和色谱的肺癌细胞磷酸化蛋白质组研究及其应用

**DOI:** 10.3724/SP.J.1123.2020.07041

**Published:** 2021-01-08

**Authors:** Baohui ZHANG, Chentong WANG, Miao GUO, Hua XIAO

**Affiliations:** 微生物代谢国家重点实验室, 上海交通大学生命科学技术学院, 上海 200240; State Key Laboratory of Microbial Metabolism, School of Life Sciences and Biotechnology, Shanghai Jiao Tong University, Shanghai 200240, China; 微生物代谢国家重点实验室, 上海交通大学生命科学技术学院, 上海 200240; State Key Laboratory of Microbial Metabolism, School of Life Sciences and Biotechnology, Shanghai Jiao Tong University, Shanghai 200240, China; 微生物代谢国家重点实验室, 上海交通大学生命科学技术学院, 上海 200240; State Key Laboratory of Microbial Metabolism, School of Life Sciences and Biotechnology, Shanghai Jiao Tong University, Shanghai 200240, China; 微生物代谢国家重点实验室, 上海交通大学生命科学技术学院, 上海 200240; State Key Laboratory of Microbial Metabolism, School of Life Sciences and Biotechnology, Shanghai Jiao Tong University, Shanghai 200240, China

**Keywords:** 固定化金属离子亲和色谱, 磷酸化, 蛋白质组, 癌症转移, 肺癌细胞, immobilized metal affinity chromatography (IMAC), phosphorylation, proteome, cancer metastasis, lung cancer cell

## Abstract

磷酸化是蛋白质翻译后修饰的重要形式之一,其异常往往会导致细胞内信号通路的紊乱和疾病的发生。固定化金属离子亲和色谱(IMAC)是磷酸化肽段的高效富集技术,在磷酸化蛋白质组研究方面应用广泛。该研究以金属钛离子(Ti^4+^)螯合IMAC材料(Ti^4+^-IMAC)为载体,进行磷酸化肽段富集。比较了10 μm Ti^4+^-IMAC通过振荡法和固相萃取法(SPE)富集磷酸肽的效果,发现振荡法可以富集到更多的磷酸肽;对比了两种尺寸(10 μm和30 μm)Ti^4+^-IMAC在磷酸化肽段富集中的差异,发现小尺寸材料富集效果更佳。进一步采用优化的策略比较了不同转移能力肺癌细胞的磷酸化蛋白质组,免标记定量蛋白质组学结果表明,优化的Ti^4+^-IMAC方法可以从正常的肺成纤维细胞MRC5、低转移肺癌细胞95C和高转移肺癌细胞95D中分别鉴定到510、863和1108种磷酸化蛋白质,其中317种为3组所共有。该研究共鉴定到1268种磷酸化蛋白质上的7560个磷酸化位点,其中1130个为差异磷酸化位点,文献报道显示部分异常表达的激酶与癌症转移密切相关。通过生信对比分析发现,异常表达的磷酸化蛋白质主要与细胞侵袭、迁移和死亡等细胞迁移方面的功能有关。通过优化磷酸化肽富集策略,初步阐明了磷酸化蛋白质网络的异常与肺癌转移之间的相关性,该方法有望用于肺癌进展相关的磷酸化位点、磷酸化蛋白质及其信号通路研究。

## Abstract (en)

Phosphorylation is one of the most important post-translational modifications in proteins. It plays a key role in numerous cellular processes, including signal transduction, cell proliferation, and intercellular communication. More than 30% of the cellular proteins are phosphorylated at a given time. However, dysregulation of phosphorylated proteins usually leads to a disorder in the intracellular signaling pathways and the onset of various diseases, especially cancer. Cell proliferation and metastasis are the major manifestations of cancer progression, and these might be affected by the protein phosphorylation levels. Clinically, cancer usually metastasizes at the middle and late stages, affecting other organs beyond primary lesion. This poses significant challenges in cancer treatment and prognosis. Consequently, comparing the phosphorylated proteomes of cells with different metastatic capabilities is helpful in studying the role of protein phosphorylation in cancer metastasis and progression. The human low metastatic lung cancer cell line 95C and high metastatic lung cancer cell line 95D are two of the four sublines isolated from human lung giant cell carcinoma cell line (PLA-801) by the single-cell cloning technique. These are ideal models for studying tumor metastasis and non-small cell lung cancer. MRC-5 cell line was obtained from a 14 week old fetal normal lung tissue. Quantitative analysis of the proteome and phosphorylated proteome in these normal lung cells and lung cancer cells with different metastatic capacities can identify key pathways and regulatory proteins associated with lung cancer metastasis and progression. Immobilized metal affinity chromatography (IMAC) is an efficient technique for the enrichment of phosphopeptides and has been widely used for phosphoproteome research. Metal ions (such as Ti^4+^) are immobilized on the substrate by chelation, and phosphopeptides can be selectively adsorbed under acidic conditions and eluted under alkaline conditions. IMAC can enrich phosphate groups at different amino acid sites with high specificity. In this study, Ti^4+^was chelated onto Ti^4+^-IMAC material, which was used to enrich phosphopeptides for phosphoproteome research. Two enrichment methods, namely, the vortexing method and solid phase extraction (SPE) method, were first compared for the enrichment of phosphopeptides using 10 μm Ti^4+^-IMAC. Phosphopeptides were highly enriched using the vortexing method. Following this, two sizes of Ti^4+^-IMAC material (10 μm and 30 μm) were compared to determine the efficiency of phosphopeptide enrichment. Enrichment efficiency was superior with the smaller-sized material. Therefore, the small-size Ti^4+^-IMAC material was selected for the proteomics research of lung cell phosphorylation. The optimized strategy was further used to compare the phosphoproteomes of the lung cancer cells with different metastatic abilities. Label-free quantification proteomics demonstrated that 510, 863, and 1108 phosphorylated proteins were identified from normal lung fibroblasts (MRC-5), low metastatic lung cancer cells (95C), and high metastatic lung cancer cells (95D), respectively, using the optimized Ti^4+^-IMAC method. Among them, 317 phosphorylated proteins were shared among the three groups. The protein phosphorylation level increased significantly with increasing cellular metastatic capacity. In our study, 7560 phosphorylation sites were identified on 1268 phosphorylated proteins, among which 1130 phosphorylation sites were differentially expressed. Some abnormally expressed kinases and their phosphorylation levels are closely associated with malignant cell proliferation. Comparative bioinformatics analysis showed that dysregulated phosphoproteins were mainly related to cell migration functions, such as cell invasion, migration, and death. These abnormally expressed phosphorylated proteins and phosphorylation sites could be further validated and studied for lung cancer metastasis. Our study demonstrates that Ti^4+^-IMAC is a powerful tool for conducting cancer metastasis-related phosphoproteome research. By optimizing the phosphopeptide enrichment strategy, our data preliminarily clarified the correlation between the abnormality of the phosphoprotein network and lung cancer metastasis. This is expected to be useful for studying phosphorylation sites, phosphorylated proteins, and their signaling pathways related to lung cancer progression.

在生命活动的调解中,蛋白质通过改变自身的丰度、定位、降解和翻译后修饰(PTM)来实现催化、免疫和细胞增殖等各项功能^[1]^。磷酸化是常见的蛋白质翻译后修饰形式^[2]^,在特定时间内,超过30%的蛋白质会发生磷酸化或以磷酸化形式呈现^[3]^。固定化金属离子亲和色谱(IMAC)是一种高效的磷酸化肽段富集技术^[4]^,金属离子(如Ti^4+^、Fe^3+^和Ga^3+^)^[5,6]^通过螯合固定在基质上,在酸性条件下吸附磷酸化肽段,并在碱性条件下洗脱^[7]^。该方法具有很高的特异性,能够富集不同氨基酸位点上的磷酸基团。新型钛离子螯合IMAC材料(Ti^4+^-IMAC)具有高选择性和高稳定性^[8]^。通过使用Ti^4+^-IMAC材料,Giansanti等^[9]^从Jurkat T细胞中分离鉴定到了30000多种特征磷酸化肽段。

蛋白质的磷酸化会影响细胞增殖、转移等特性^[10]^,而癌症的转移又是癌症进展和恶化的主要表现。在临床上,癌症通常会在中晚期发生转移,影响原发灶以外的其他脏器,给治疗和预后带来极大挑战。以肺癌为例,其常见的转移部位包括肝脏、大脑、骨骼和淋巴等,而相应的生物学机制尚不十分清楚^[11]^。因此,比较不同转移能力细胞的磷酸化蛋白质组有助于研究蛋白质磷酸化在癌症转移和进展中的作用。

人低转移肺癌细胞95C和人高转移肺癌细胞95D是通过单细胞克隆技术从人肺巨细胞癌细胞系(PLA-801)中分离得到的4个亚系(A、C、D、E株)中的两株。皮下接种裸鼠后,D株(95D)自发转移的发生率较高,A、E株中等,而C株(95C)较低,95D和95C是研究肿瘤转移和非小细胞肺癌的理想模型。MRC5是贴壁、梭型、成纤维肺细胞,从14周胎儿正常肺组织中获得。研究这些正常肺细胞和不同转移能力的肺癌细胞中的蛋白质组和磷酸化蛋白质组,将有助于发现与肺癌转移进展相关的关键通路和调控蛋白^[11]^。

本文在现有Ti^4+^-IMAC方法的基础上,比较两种尺寸(10 μm和30 μm)Ti^4+^-IMAC在磷酸肽富集中的差异,并对比了10 μm Ti^4+^-IMAC通过振荡法和固相萃取法(SPE)富集磷酸化肽的效果,采用优化的策略研究了3种转移能力不同的肺细胞系(MRC5、95C和95D),通过对不同转移能力肺细胞的蛋白质组和磷酸化蛋白质组进行比较分析,对差异蛋白质进行生信分析,揭示了差异表达的磷酸化位点和磷酸化肽段,从而发掘肺癌转移相关的磷酸化蛋白质。

## 1 实验部分

### 1.1 仪器、试剂与材料

纳升液相色谱(Easy-nLC1000)和轨道离子阱质谱仪(LTQ-Orbitrap XL)购于赛默飞公司(美国)。电热鼓风干燥箱购于上海恒科学仪器有限公司(中国),超声波清洗器购于昆山市超声波仪器有限公司(中国), 5424离心机和离心浓缩仪购于艾本德公司(美国),多功能酶标仪购于GE公司(美国)。

蛋白质酶抑制剂和磷酸酶抑制剂购于罗氏公司(瑞士), Ti^4+^-IMAC(~10 μm)和Ti^4+^-IMAC(~30 μm)购于百灵威科技有限公司(中国),蛋白质浓度测定(BCA)试剂盒购于赛默飞公司(美国), MEM基本培养基和RPMI1640基本培养基购于Gibco公司(美国)。

### 1.2 模式磷酸化肽段样本的制备

对牛血清白蛋白(BSA)和*α*-酪蛋白(*α*-casein)分别进行酶解,并按照质量比200:1的比例混合,制备模式磷酸化肽段样本。

### 1.3 细胞培养和细胞蛋白质酶解

MRC5使用MEM培养基培养,95C和95D细胞使用RPMI 1640培养基培养(10%胎牛血清,1×青霉素/链霉素溶液)。所有细胞在含5% CO_2_的37 ℃培养箱中培养,待细胞长到对数生长期后期传代,胰酶37 ℃消化数分钟,传代密度控制在细胞增殖最快的状态。取长势良好的细胞,300×g下离心5 min,弃上清,收集细胞沉淀,用含6 mol/L尿素的碳酸氢铵溶液裂解细胞,弃上层脂质部分,向澄清的细胞样本中加入蛋白酶抑制剂和磷酸酶抑制剂,得到细胞蛋白质样本。参照BCA蛋白质浓度测定试剂盒说明书测定细胞蛋白样本的浓度。取适当质量的蛋白质溶液,加入终浓度2 mmol/L二硫苏糖醇(DTT), 60 ℃水浴1 h,加入10 mmol/L碘乙酰胺(IAA),室温避光反应40 min。按照常规FASP(filter-aided sample preparation)方法进行蛋白酶解(胰酶与样品蛋白质的质量比为1:50)。

### 1.4 磷酸化肽段的富集

将30 μm的大尺寸Ti^4+^-IMAC标记为Ti^4+^-IMAC-L,将10 μm的小尺寸Ti^4+^-IMAC标记为Ti^4+^-IMAC-S,按照Ti^4+^-IMAC说明书将Ti^4+^螯合在IMAC上,并配制以下溶液:上样缓冲液(80%(v/v,下同)乙腈,6%三氟乙酸)、洗涤缓冲液1(50%乙腈,6%三氟乙酸,200 mmol/L氯化钠)、洗涤缓冲液2(30%乙腈,0.1%三氟乙酸)、洗脱缓冲液1(10%氨水)和洗脱缓冲液2(1%甲酸)。

振荡法富集磷酸化肽段方法:将冻干后的肽段样品重溶于1倍体积的上样缓冲液至终浓度为1 mg/mL,将肽段溶液与Ti^4+^-IMAC混合(材料与肽段质量比为25:1)振荡30 min, 15000×g离心去上清。加入1倍体积的洗涤缓冲液1,振荡30 min,离心弃上清,再加入1倍体积的洗涤缓冲液2洗涤两次(混合振荡15 min并离心弃上清),将盐离子充分洗去。加入150 μL洗脱缓冲液1超声15 min,再振荡15 min,离心收集洗脱液,再向材料中加入150 μL洗脱缓冲液1振荡15 min,离心收集第二次的洗脱液,将两份洗脱液合并,18000×g离心5 min,充分去除材料沉淀,真空干燥。

SPE法富集磷酸化肽段^[12]^:参考文献^[12]^制作SPE柱,肽段溶液与Ti^4+^-IMAC质量比为1:20,加入缓冲液的体积使肽段浓度保持在1 mg/mL左右。用适当转速离心,使液体全部流下时间约为10 min。加入200 μL洗涤缓冲液1,用相同转速离心至液体全部流下并重复洗涤一次,再用洗涤缓冲液2以同样方法洗涤两次。更换收集管,加入200 μL洗脱缓冲液洗涤1两次,再用100 μL洗脱缓冲液2洗脱两次。将滤出液真空干燥。

### 1.5 液相色谱-质谱分析

取细胞总蛋白质酶解后的肽段,通过ZipTipC18除盐。取2 μg除盐后的肽段上样进行基于液相色谱-质谱的蛋白质组分析,每个样品进行3次技术重复。

取500 μg各细胞总蛋白质酶解后的肽段,采用Ti^4+^-IMAC富集磷酸化肽段,除盐后上样,进行基于液相色谱-质谱的磷酸化蛋白质组分析,每个样品进行3次技术重复。

液相色谱-质谱的相关参数如下:C18反相柱(15 cm×75 μm, 3 μm, Thermo Fisher Scientific),离子源为电喷雾(ESI),质谱电喷雾毛细管电压1900 V,气体流速2.0 L/min,离子源温度为120 ℃,选择质核比为350~2000的母离子进行CID(collision-induced dissociation)二级碎裂扫描,碰撞能量为20~70 eV,动态排除0.25 min。

### 1.6 数据分析

Proteome Discoverer(PD,版本1.3)搜库:Homo sapiens数据库(版本2017_11_28, 20244条序列),胰酶消化,母离子质量误差值为15 ppm(15×10^-6^),漏切位点最大值设置为2;蛋白质和肽段鉴定假阳性率(FDR)小于1%,蛋白质鉴定标准为至少检测到两个特征肽段。

MaxQuant(版本1.6.7.0)搜库:数据库、消化酶、漏切位点最大值、固定修饰、可变修饰等参数与PD搜库的设置相同。母离子质量误差值设置为20 ppm(20×10^-6^),主要搜索肽段误差值为4.5 ppm(4.5×10^-6^)。蛋白质鉴定标准为至少检测到两个特征肽段,且肽段长度大于或等于7个氨基酸,设置蛋白质及二级谱图数量(peptide-spectrum matches, PSM)的假阳性率为0.01。用Perseus软件进行数据分析^[13]^,筛选肽段及位点等,筛选出符合要求的总蛋白质和磷酸化蛋白质、总肽段和磷酸化肽段以及磷酸化位点(位置可能性>0.75)。

免标记定量蛋白质组学分析以四分位法对iBAQ(intensity-based absolute quantification)值进行归一化。针对磷酸化位点的定量分析,我们首先以在一组中至少有两个以上强度值不为零的标准筛选磷酸化位点,用*t*-test检验法计算*p*值。然后,筛选有显著差异的(*p*-value<0.05)磷酸化位点,并计算它们的上下调倍数(fold change, FC)。若FC≥1.5或≤0.66且*p*-value<0.05,即为显著上调或下调的磷酸化位点。

对蛋白质组和磷酸化蛋白质组进行生物学分析,利用GO(Gene Ontology)分析、KEGG(Kyoto Encyclopedia of Genes and Genomes)通路分析等探索蛋白质功能。对定量数据进行IPA(Ingenuity Pathway Analysis, Qiagen)途径分析,包括经典通路、疾病和生物学功能分析等,并对磷酸化蛋白质组和蛋白质组进行比较分析。

## 2 结果与讨论

### 2.1 Ti^4+^-IMAC-S通过SPE和振荡法富集模式磷酸化肽段的特异性比较

为优化富集方法,采用模式磷酸化肽段比较了SPE^[8]^和振荡法对Ti^4+^-IMAC-S富集效果的影响。分别选取200 μg和400 μg为肽段的富集起始量,在相同条件下酶解模式磷酸化肽段。然后利用SPE和振荡法对相同起始量的肽段进行磷酸化肽的富集,PD的搜库结果表明,与SPE相比,振荡法可以富集到更多的磷酸化肽段(见表1)。

**表 1:** Ti^4+^-IMAC-S通过SPE和振荡法富集磷酸化肽段的特异性

Enrichment method	Protein initiation amount/μg	Number of identified phosphopeptides	Number of identified peptides	Percent of phosphopeptides/ %	Phosphorylation PSM	PSM	Percent of phosphorylation PSM/%	Number of phosphorylation sites
PE	200	21	40	52.50	53	75	70.67	31
	400	50	114	43.86	197	333	59.16	88
Vortexing	200	31	65	47.69	55	96	57.29	43
	400	62	112	55.36	192	272	70.59	88

Ti^4+^-IMAC-S: 10 μm Ti^4+^-immobilized metal affinity chromatography; PSM: peptide spectrum matches.

在富集效率方面,振荡法和SPE相差不大。SPE可用于样品量较大的研究^[12]^。同时发现,样品起始用量越大,所检测到的磷酸化肽段数就越多。在起始蛋白质用量为200 μg和400 μg的条件下,振荡法中材料与肽段的振荡混合时间为30 min,接触时间较长,磷酸化肽段被材料吸附的概率增大,因此Ti^4+^-IMAC-S用振荡法富集到的磷酸化肽段较多。

### 2.2 Ti^4+^-IMAC-S与Ti^4+^-IMAC-L在95C细胞磷酸化肽段富集中的比较分析

用振荡法,尺寸不同的Ti^4+^-IMAC-S和Ti^4+^-IMAC-L分别对95C细胞磷酸化肽段进行富集。Ti^4+^-IMAC-L在溶液中较分散,无法通过振荡后离心的方法使固液分离,因此采用SPE进行磷酸化肽段的富集^[12]^。磷酸肽的分析结果(PD搜库)如表2所示,与Ti^4+^-IMAC-L相比,Ti^4+^-IMAC-S富集到的磷酸化肽段的数量更多,特异性更高。

**表 2:** Ti^4+^-IMAC-S和Ti^4+^-IMAC-L富集95C细胞磷酸化肽段的特异性

Material	Number of identified phosphopeptides	Number of identified peptides	Percent of phosphopeptides/ %	Phosphorylation PSM	PSM	Percent of phosphorylation PSM/%	Number of phosphorylation sites	Number of phosphoproteins
Ti^4+^-IMAC-S	1644	2258	72.81	3297	4016	82.10	1804	914
Ti^4+^-IMAC-L	1002	2072	48.36	2006	3119	64.32	1095	626

Ti^4+^-IMAC-L: 30 μm Ti^4+^-immobilized metal affinity chromatography.

### 2.3 不同转移能力肺细胞蛋白质组和磷酸化蛋白质组的比较分析

为探究细胞蛋白质组和磷酸化蛋白质组之间的联系,我们进行了MRC5、95C和95D的细胞蛋白质组和磷酸化蛋白质组分析。基于上述方法优化结果,我们选用Ti^4+^-IMAC-S(振荡法)进行了细胞的磷酸化肽段富集。取相同质量肽段样品进行LC-MS/MS分析(3次技术重复),实验结果采用Maxquant软件进行分析。

2.3.1 肺细胞蛋白质组定量分析

图1a为MRC5、95C和95D细胞系全蛋白质组的韦恩图,3种细胞系的3次技术重复中共鉴定到1262种蛋白质,3种细胞系全蛋白质数目较为接近,分别鉴定到992、1102、1182种蛋白质,其中共有的蛋白质达到840种,占所有检测到蛋白质的66.6%。

**图 1:**
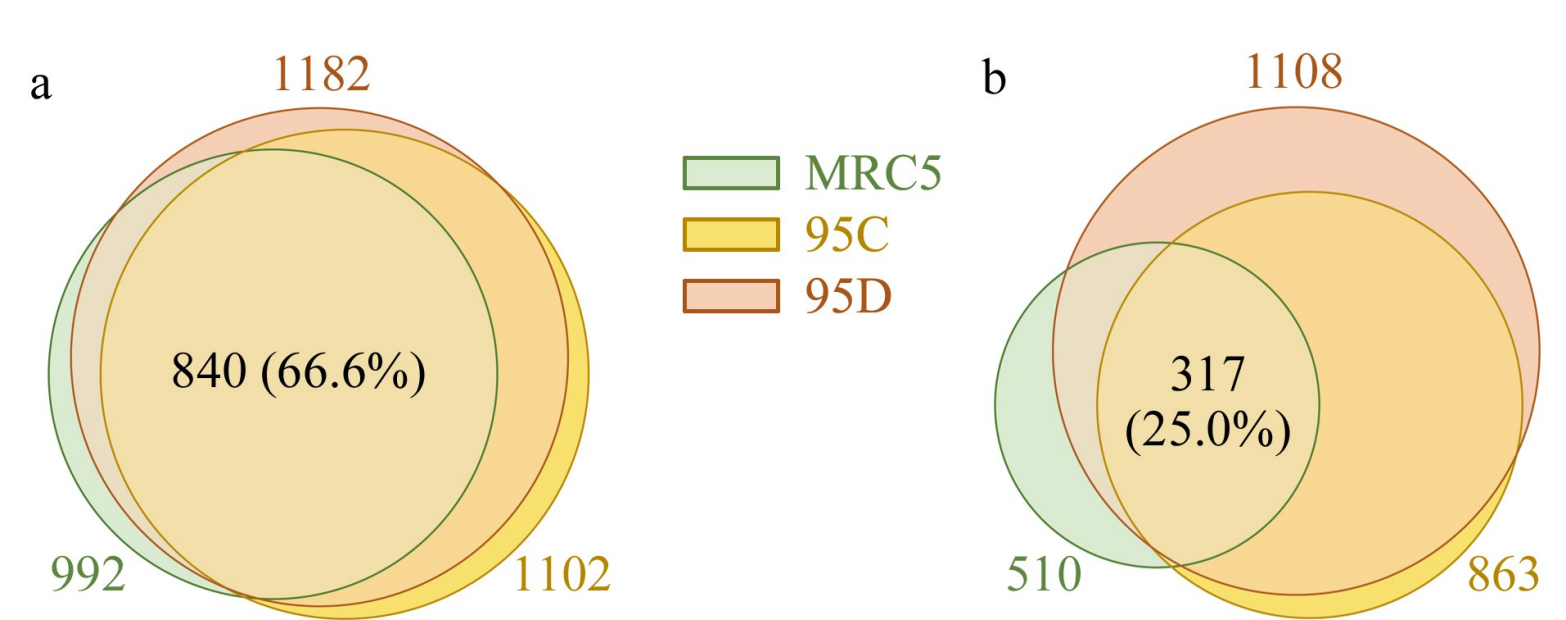
MRC5、95C和95D细胞系(a)全蛋白质和(b)磷酸化蛋白质的韦恩图

与正常肺细胞MRC5相比,95C中有325个蛋白质上调,292个蛋白质下调(见图2a)。与MRC5相比,95C和95D中上调的蛋白质数目依次增加,细胞活性增强,转移能力和增殖能力也随之越强。

**图 2:**
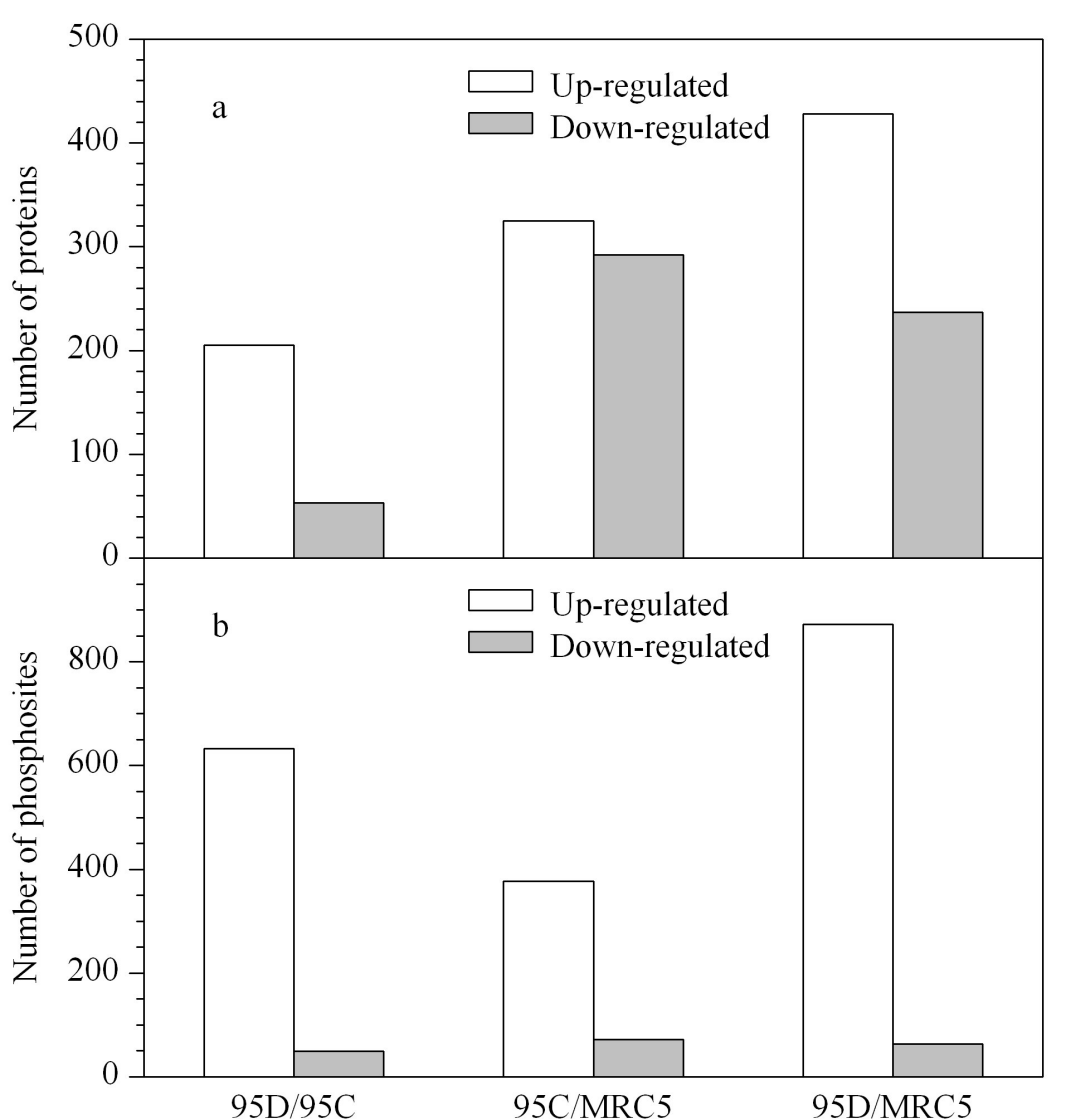
MRC5、95C和95D细胞差异表达的(a)蛋白质和(b)磷酸化位点

2.3.2 肺细胞磷酸化蛋白质组定量分析

与全蛋白质相比,3种细胞系共有的磷酸化蛋白质种类比例显著下降。如图1b所示,MRC5、95C和95D细胞系分别富集到510、863和1108种磷酸化蛋白质,只有317种磷酸化蛋白质是3种细胞系共有的,仅占25.0%。3种细胞系中较大差异的磷酸化蛋白质能够反映细胞的更多动态信息,为研究细胞转移等特征提供了依据。研究发现,高转移的肺癌细胞有更多的蛋白质发生了磷酸化修饰,并且蛋白质上的磷酸化位点也更多(见表3)。95D中含有7到11个磷酸化位点、13到15个磷酸化位点的磷酸化蛋白质数量比95C和MRC5明显增多。

**表 3:** 磷酸化蛋白质位点数频数表

Cell line	Phosphosite number per protein																
	1	2	3	4	5	6	7	8	9	10	11	12	13	14	15		16
MRC5	331	88	30	15	8	2	1	1	0	2	0	0	0	0	1		0
95C	502	169	59	21	12	13	7	2	2	0	0	1	2	0	0		0
95D	658	190	90	42	27	11	14	4	5	3	1	0	3	3	2		0

Numbers in bold indicate that 95D group has more proteins with 7-11 or 13-15 phosphosites than that in MRC5 and 95C group.

3种细胞系中共检测到7560个磷酸化位点,其中定量到1983个磷酸化位点。显著变化的磷酸化位点的数量如图2b所示,肺癌细胞比正常肺细胞磷酸化位点多,其中高转移能力肺癌细胞95D的磷酸化位点数最多。蛋白质的磷酸化修饰是控制细胞代谢的调节方式,由于MRC5细胞为肺成纤维细胞,而95C和95D分别为低转移肺癌细胞和高转移肺癌细胞,因此这些结果表明,癌细胞较正常细胞代谢旺盛,分裂能力更强。虽然3种细胞的蛋白质种类及含量差异较小,但由于蛋白质的磷酸化水平发生变化,因此起调节作用和信号转导功能的磷酸化蛋白质和磷酸化位点差异较大,说明蛋白质磷酸化可能在癌症转移过程中起重要作用。

对3种细胞系中富集到的磷酸化肽段及磷酸化位点进行分析发现,三磷酸化肽段(3-P)在95D中数量最多,占比最高(见图3a)。图3b显示了丝氨酸、苏氨酸和酪氨酸上磷酸化的数量及比例,结果表明,丝氨酸磷酸化在转移能力越高的细胞中含量略有升高。

**图 3:**
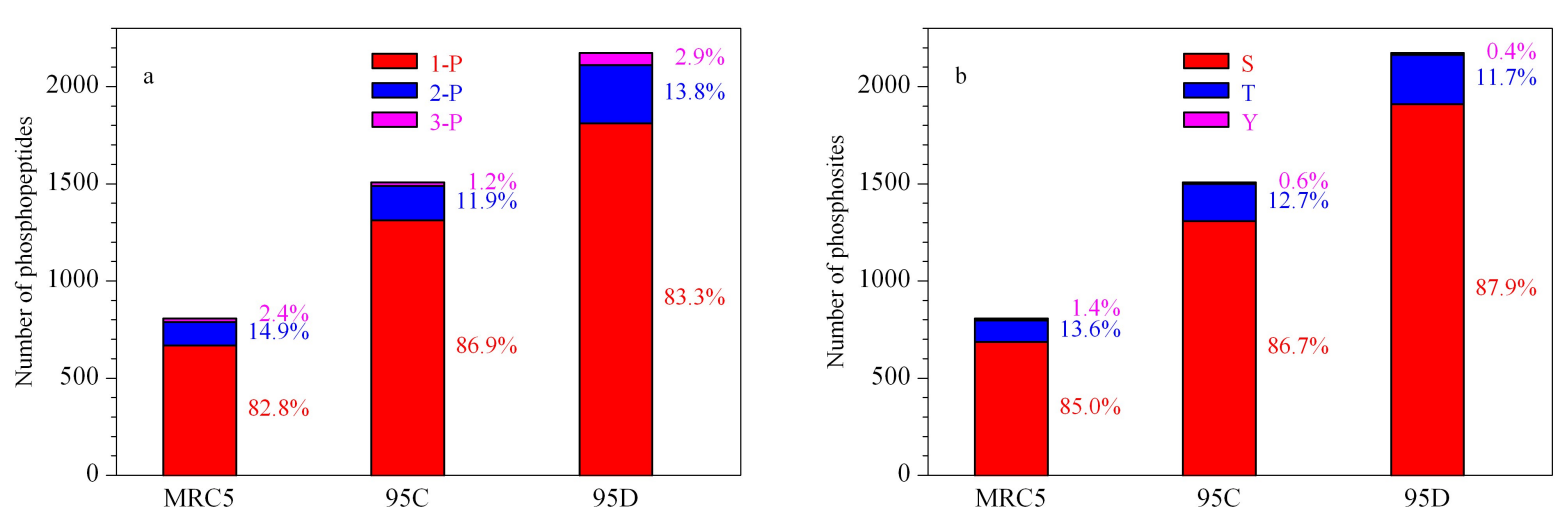
(a)单、多磷酸化肽段和(b)磷酸化位点的数量及比例

2.3.3 磷酸化蛋白质组的生物信息学分析

对1130个异常表达的磷酸化位点所对应的磷酸化蛋白质进行GO分析(见表4)。结果表明,这些差异磷酸化蛋白质参与的生物学过程集中在代谢和RNA加工相关的过程,所属的细胞成分主要集中在细胞核,具有的分子功能表现为与蛋白质、核酸均有结合。

**表 4:** 异常表达磷酸化位点对应磷酸化蛋白质的GO分析

GO catalogue	Term	Gene percent/%	False discovery rate
Biological process	mRNA metabolic process	14.84	2.20E-29
	RNA processing	12.97	7.98E-28
	mRNA processing	16.67	5.06E-25
	nucleic acid metabolic process	6.50	5.58E-25
	RNA splicing	17.90	1.10E-24
	nucleobase-containing compound metabolic process	6.13	2.34E-24
	organelle organization	6.90	3.37E-23
	cellular component organization or biogenesis	5.73	5.65E-23
	regulation of metabolic process	5.33	4.28E-22
	regulation of organelle organization	9.87	2.77E-21
Cell component	nuclear part	7.89	5.86E-59
	nuclear lumen	8.04	4.66E-56
	nucleoplasm	8.39	1.72E-51
	intracellular part	4.43	1.78E-50
	nucleus	6.11	2.14E-48
	intracellular	4.35	1.11E-47
	intracellular organelle	4.72	1.77E-46
	organelle	4.66	2.10E-45
	cytosol	6.84	3.44E-44
	intracellular non-membrane-bounded organelle	7.37	3.45E-42
Molecular function	protein binding	5.45	2.23E-25
	binding	4.39	5.64E-23
	cytoskeletal protein binding	10.09	9.23E-17
	RNA binding	10.00	8.07E-16
	enzyme binding	6.92	1.94E-15
	heterocyclic compound binding	5.07	2.09E-12
	nucleic acid binding	5.70	5.80E-12
	protein domain specific binding	9.63	6.60E-12
	organic cyclic compound binding	5.00	8.06E-12
	kinase binding	9.44	7.03E-11

磷酸化位点定量分析得到了73个在MRC5、95C、95D中依次上调的磷酸化位点,对这些磷酸化位点对应的磷酸化蛋白质进行GO分析,有助于探究这些在转移能力较强的肺细胞中上调的磷酸化位点与细胞转移和癌变的关系。分析结果表明,在假阳性率最低的前10个生物学过程中,与细胞正调控有关的过程有4个,与细胞周期、有丝分裂有关的过程有3个。与所有异常表达的1130个磷酸化位点所对应的RNA有关的生物过程不同,这些磷酸化蛋白质偏向于与细胞癌变有关;对于细胞成分而言,大多数磷酸化蛋白质都集中在细胞核部分,与蛋白质组相同;分子功能分析中,与激酶结合的占3个,置信度较高,且与DNA结合置信度及位点所对应的蛋白质所占比例均较大。因为MRC5、95C、95D依次上调的磷酸化位点所对应的蛋白质主要参与了细胞分裂周期的调节及DNA的结合,所以对磷酸化位点进行定量分析是研究细胞转移及癌变过程的一个重要切入点,可以为后续机理或生物标志物研究奠定基础,为癌症等疾病诊断提供依据。

2.3.4 激酶及其磷酸化位点分析

全蛋白质组中激酶表达情况与磷酸化蛋白质组有一定关联,全蛋白质组共鉴定到43种激酶,其中28种激酶在3种细胞中有显著变化,如表5所示。

**表 5:** 全蛋白质组显著调节的激酶相关蛋白质

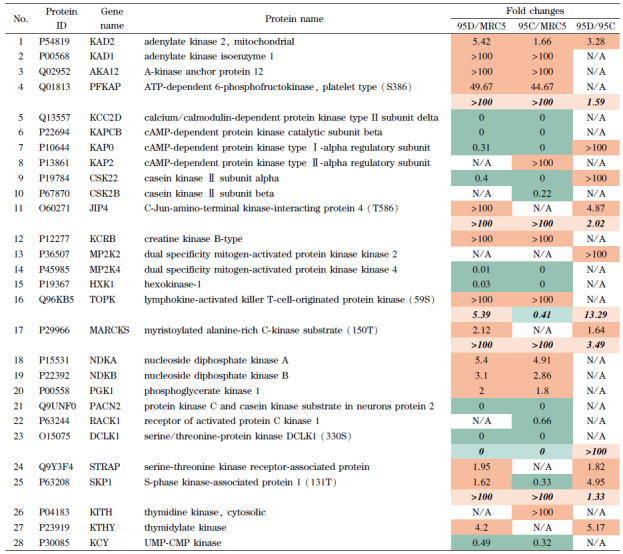

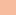
 up-regulated protein; 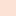 up-regulated phosphorylation sites; 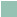 down-regulated protein; 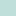 down-regulated phosphorylation sites. Normal font: proteins associated with significantly regulated kinases revealed by proteome analysis; bold font: proteins associated with significantly regulated kinases revealed by phosphoproteome analysis; italic bold font: fold change of phosphorylation sites on corresponding proteins. N/A: no significant differences.

其中,6种激酶在磷酸化肽富集之后的位点分析中同样出现显著性变化(粗体显示),分别是ATP依赖6磷酸果糖激酶(PFKAP)、C-Jun-氨基末端激酶相互作用蛋白4(JIP4)、T淋巴细胞活化的杀伤细胞来源蛋白激酶(TOPK)、富含肉豆蔻碱的C激酶底物(MARCKS)、丝氨酸/苏氨酸蛋白激酶(DCLK1)和S期激酶相关蛋白1(SKP1),其磷酸化位点上下调的倍数显示在蛋白质的倍数下方(斜体加粗)。对于这28个差异激酶,经文献检索,选取其中8个分析其典型激酶调节特征。

1. 腺苷酸激酶(KAD):是催化腺嘌呤核苷酸(AMP/ADP/ATP)相互转化的磷酸转移酶,在维持细胞能量平衡中起重要作用,在95C和95D细胞中依次呈现过表达趋势。研究^[14]^发现,KAD2在肺腺癌患者肺癌组织中过表达,且KAD2的下调可抑制人肺腺癌细胞的增殖、迁移和侵袭,诱导细胞凋亡和自噬。

2. 激酶A锚定蛋白12(AKA12):仅在95C和95D细胞中被检测到,在MRC5细胞中未鉴定到。研究^[15]^发现,AKA12是介导蛋白激酶A和蛋白激酶C亚细胞分区的锚定蛋白质,与细胞内β肾上腺素受体信号调节相关。

3. PFKAP:与糖酵解相关^[16]^,在癌细胞中高表达。值得注意的是,该酶的磷酸化水平在癌细胞中也显著升高,说明PFKAP在癌细胞中代谢活力强。

4. 肌酸激酶(KCRB):能够可逆催化ATP的合成,在能量需求大的细胞中高表达,也有大量研究表明KCRB为潜在肿瘤标志物^[17]^。

5. JIP4:又叫人肺致癌基因6蛋白,C-Jun是原癌基因,在多种癌症中表达显著增强^[18]^,在本文实验数据中,JIP4及其磷酸化位点(T586)在癌细胞中表达增强。

6. TOPK:在癌细胞中高表达,研究^[19]^表明TOPK在白血病和骨髓瘤等高增殖肿瘤中过表达,在肿瘤发生和转移中起关键作用。

7. MARCKS及其磷酸化:在癌细胞中高表达,研究^[20]^发现MARCKS可以调节胆道癌细胞的转移。

8. SKP1:研究^[21]^发现,其在56.3%的非小细胞肺癌中过表达,且预后不良时其表达升高;在95D细胞中高表达,磷酸化水平显著提高,现象与文献^[21]^一致。

我们鉴定到的异常表达的激酶中大部分已被证实与细胞癌变及转移密切相关,另有部分异常表达的激酶尚未见报道,可作候选蛋白质,验证其成为生物标志物的可能性。因此,全蛋白质水平的激酶调节对癌症进展和后续磷酸化蛋白质组的研究都具有重要意义。

2.3.5 蛋白质组与磷酸化蛋白质组对比分析

将显著变化的蛋白质与磷酸化蛋白质进行了IPA分析。在疾病和生物功能方面,蛋白质组显示了与DNA、细胞周期有关的上调,而与RNA、细胞死亡有关的下调,并且肺癌进展有关的蛋白质上调。磷酸化蛋白质组显示了与死亡相关、细胞生长相关的蛋白质下调,迁移侵袭、DNA修复有关的蛋白质上调。磷酸化蛋白质组的对比分析发现,95D/95C、95C/MRC5、95D/MRC5有相同的变化趋势,即MRC5、95C、95D依次上调或下调,且与细胞增殖和转移有关的磷酸化蛋白质上调。与蛋白质组相比,磷酸化蛋白质组更能反映细胞的转移能力的差异。

本研究鉴定到的Histone H1.5(H15)、真核翻译起始因子4B(IF4B)、富含十四烷基的富含丙氨酸的C激酶底物(MARCS)和神经母细胞分化相关蛋白(AHNAK)等蛋白质的磷酸化位点表达量变化也与肺癌进展正相关^[22]^,提示其在肺癌转移中的潜在功能。

## 3 结论

与大尺寸的Ti^4+^-IMAC-L相比,Ti^4+^-IMAC-S在富集磷酸化肽段的数量和特异性上更优。与SPE相比,振荡法由于样品与Ti^4+^-IMAC-S材料接触的时间较长,富集到的磷酸化肽段数量更多。将Ti^4+^-IMAC-S的振荡法应用于不同转移能力的肺细胞磷酸化蛋白质组学研究中发现,3种细胞系的全蛋白质水平差异较小,而磷酸化蛋白质水平差异较大。随着细胞转移能力的增加,蛋白磷酸化水平也显著增加。其中95C和95D上调的磷酸化位点对应的蛋白质与细胞周期、细胞增殖显著相关。一些异常表达的激酶及其磷酸化程度与细胞的恶性增殖有关。异常表达的磷酸化蛋白质主要与细胞侵袭、迁移和死亡等细胞迁移方面的功能有关,后续研究可对这些异常表达的磷酸化蛋白质和磷酸化位点进行肺癌转移等功能的验证。本研究充分说明基于Ti^4+^-IMAC的亲和色谱技术是开展癌症转移相关研究的强有力工具。
